# Angiogenesis, a key point in the association of gut microbiota and its metabolites with disease

**DOI:** 10.1186/s40001-024-02224-5

**Published:** 2024-12-23

**Authors:** Yan Wang, Mingshuai Bai, Qifan Peng, Leping Li, Feng Tian, Ying Guo, Changqing Jing

**Affiliations:** 1 Department of Gastrointestinal Surgery, Shandong Provincial Hospital, Shandong University, Jinan, 250021 Shandong China; 2 Department of Gastrointestinal Surgery, Shandong Provincial Hospital Affiliated to Shandong First Medical University, Jinan, 250021 Shandong China; 3 Department of Breast and Thyroid Surgery, Shandong Provincial Hospital Affiliated to Shandong First Medical University, Jinan, 250021 Shandong China

**Keywords:** Intestinal microbiota, Metabolites, Angiogenesis, Lymphangiogenesis

## Abstract

The gut microbiota is a complex and dynamic ecosystem that plays a crucial role in human health and disease, including obesity, diabetes, cardiovascular diseases, neurodegenerative diseases, inflammatory bowel disease, and cancer. Chronic inflammation is a common feature of these diseases and is closely related to angiogenesis (the process of forming new blood vessels), which is often dysregulated in pathological conditions. Inflammation potentially acts as a central mediator. This abstract aims to elucidate the connection between the gut microbiota and angiogenesis in various diseases. The gut microbiota influences angiogenesis through various mechanisms, including the production of metabolites that directly or indirectly affect vascularization. For example, short-chain fatty acids (SCFAs) such as butyrate, propionate, and acetate are known to regulate immune responses and inflammation, thereby affecting angiogenesis. In the context of cardiovascular diseases, the gut microbiota promotes atherosclerosis and vascular dysfunction by producing trimethylamine N-oxide (TMAO) and other metabolites that promote inflammation and endothelial dysfunction. Similarly, in neurodegenerative diseases, the gut microbiota may influence neuroinflammation and the integrity of the blood–brain barrier, thereby affecting angiogenesis. In cases of fractures and wound healing, the gut microbiota promotes angiogenesis by activating inflammatory responses and immune effects, facilitating the healing of tissue damage. In cancer, the gut microbiota can either inhibit or promote tumor growth and angiogenesis, depending on the specific bacterial composition and their metabolites. For instance, some bacteria can activate inflammasomes, leading to the production of inflammatory factors that alter the tumor immune microenvironment and activate angiogenesis-related signaling pathways, affecting tumor angiogenesis and metastasis. Some bacteria can directly interact with tumor cells, activating angiogenesis-related signaling pathways. Diet, as a modifiable factor, significantly influences angiogenesis through diet-derived microbial metabolites. Diet can rapidly alter the composition of the microbiota and its metabolic activity, thereby changing the concentration of microbial-derived metabolites and profoundly affecting the host's immune response and angiogenesis. For example, a high animal protein diet promotes the production of pro-atherogenic metabolites like TMAO, activating inflammatory pathways and interfering with platelet function, which is associated with the severity of coronary artery plaques, peripheral artery disease, and cardiovascular diseases. A diet rich in dietary fiber promotes the production of SCFAs, which act as ligands for cell surface or intracellular receptors, regulating various biological processes, including inflammation, tissue homeostasis, and immune responses, thereby influencing angiogenesis. In summary, the role of the gut microbiota in angiogenesis is multifaceted, playing an important role in disease progression by affecting various biological processes such as inflammation, immune responses, and multiple signaling pathways. Diet-derived microbial metabolites play a crucial role in linking the gut microbiota and angiogenesis. Understanding the complex interactions between diet, the gut microbiota, and angiogenesis has the potential to uncover novel therapeutic targets for managing these conditions. Therefore, interventions targeting the gut microbiota and its metabolites, such as through fecal microbiota transplantation (FMT) and the application of probiotics to alter the composition of the gut microbiota and enhance the production of beneficial metabolites, present a promising therapeutic strategy.

## Introduction

Lymphatic and blood vessels are critical components of the human circulatory system, and their formation is associated with a variety of diseases, including cancer [1–3], inflammatory skin diseases [4], kidney diseases [5], and cardiovascular diseases [6]. The gut microbiota is a vital component of the human digestive system, playing a pivotal role in health and disease. *Firmicutes* (such as the *genus Clostridium*) and *Bacteroidetes* (such as the *genus Bacteroides*) constitute 90% of the gut microbiota [7]. The varied physicochemical characteristics of the small intestine and colon—including pH levels, oxygen availability, and the presence of antimicrobial substances—result in a layered distribution of the gut microbiota throughout the digestive system [8]. In addition,research indicates that lower socioeconomic status is associated with unhealthy lifestyles, poor dietary choices, and suboptimal environmental conditions, which can lead to decreased gut microbial diversity and a higher risk of multi-drug resistant organism (MDRO) colonization [9]. The colonization of gut microbiota plays a crucial role in the development of intestinal microvascular and lymphatic networks during early life [10]. Based on a germ-free mouse model, the researchers discovered that the influence of intestinal flora on small intestinal vascular remodeling may be associated with the activation of the coagulation pathway, the expression and activation of transcription factors (TF) in intestinal epithelial cells promote the dilation of villous capillaries through protease-activated receptor-1 (PAR-1) dependent signaling [11]. The regulation of intestinal barrier function by intestinal flora is both dynamic and balanced [12].The metabolic byproducts of gut microbiota enter the liver via the portal vein circulation, where they regulate angiogenesis and lymphangiogenesis within the liver microcirculation [13]. This regulatory effect is mediated by Paneth cells [14–16]. Intestinal macrophages promote the maturation of lymphatic vessels by sensing microbial patterns and producing cytokines such as VEGF-C. Chylomicron defects have also been observed in germ-free mice; however, normal chylomicron structure can be restored through conventional treatment, specifically by exposing germ-free mice to a normal microbial environment [17]. The metabolites of the gut microbiota play an important role in maintaining the growth, function, and metabolism of skeletal muscle. During the aging process, the production of D-malic acid (DMA) increases, inhibiting angiogenesis and leading to muscle atrophy during aging [18]. Increasing evidence suggests that signals derived from gut microbiota may also influence the vasculature of distant organ systems, including the microvasculature of the brain [19] and eyes [20]. Previous studies have highlighted the crucial role of the gut microbiome and its metabolites in the onset and progression of various diseases, including obesity [21, 22] diabetes [23], cardiovascular diseases [24, 25], systemic inflammation [26], cancer [27], and aging [28]. Recent studies indicate that the impact of gut microbiota and their metabolites on these diseases may be mediated through their influence on lymphangiogenesis and angiogenesis [10, 29–32]. Based on a germ-free mouse model, researchers observed that the experimental group of mice, which were gavaged with feces from CRC patients, had a higher cancer incidence compared to the control group. The fecal gut microbiome abundance in the experimental group was reduced, and there was an upregulation of gene expression related to tumor angiogenesis and cell proliferation, along with an increase in the expression of inflammatory factors in the gut. This suggests that the gut microbiome in colorectal cancer may have a tumor-promoting effect, while the absence of tumor formation in the control group indicates that a normal gut microbiome does not have a tumor-promoting effect [33]. Gut microbiota and their metabolites affect the formation of blood and lymphatic vessels via various mechanisms, including the induction of chronic inflammation [34, 35], modulation of gut mucosal integrity, and the regulation of immune system functions [36, 37]. Diet can influence angiogenesis by intervening in the composition of gut microbiota and the production of metabolic products. For example, diets high in red meat and fat can increase the production of TMAO, promoting the occurrence and development of atherosclerosis in the body. In contrast, a fiber-rich diet can enhance the production of SCFAs, exerting anti-inflammatory effects in the gut, protecting the intestinal barrier, and potentially playing a protective role in inflammatory bowel disease (IBD) and colon cancer. This indicates that fecal microbiota transplantation (FMT), along with the use of oral antibiotics, probiotics, and other therapeutic interventions, can alter the composition of the gut microbiota and its metabolic byproducts, including TMAO and SCFAs. Such alterations may influence the regulation of angiogenesis and lymphangiogenesis, potentially aiding in the prevention and management of a range of diseases.

## The impact of gut microbiota and its metabolites on developmental angiogenesis.

By comparing germ-free mice with ex-germ-free animals that were colonized during or after later stages of intestinal development, researchers found that the formation of the intestinal microvascular network in adult germ-free mice was inhibited (Table 1). This developmental defect can be reversed by colonization with a complete microbiota or the single species *Bacteroides thetaiotaomicron* (*B. thetaiotaomicron*). Further comparisons between germ-free mice and transgenic mice lacking Paneth cells, which were colonized with *B. thetaiotaomicron*, revealed that the regulation of angiogenesis by the microbiota is dependent on Paneth cells [16]. This finding indicates that the gut microbiota plays a crucial role in the development of the microvascular network within intestinal villi, with Paneth cells being instrumental in coordinating the interaction between the gut microbiota and the formation of the intestinal microvascular network. [38]. Furthermore, In the germ-free (GF) mouse model, it was observed that colonization by the gut microbiota promotes the glycosylation of tissue factor (TF), activates the coagulation pathway, and increases small intestinal vascular density through the TF-protease-activated receptor 1 (PAR1) signaling pathway, thereby enhancing intestinal barrier function [11]. Under a high-fat diet, the offspring of GF pregnant mice are more prone to metabolic syndrome compared to the offspring of SPF (specific pathogen-free) pregnant mice. This is due to the short-chain fatty acids (SCFAs) in the colonic lumen of the pregnant mice, which reach the embryos through the maternal liver and bloodstream, promoting cell differentiation [39].The gut microbiota and its metabolic products play a key role in physiological angiogenesis. Recently, the concept of the gut-vascular axis has drawn attention to the role of gut microbiota dysbiosis in pathological angiogenesis [40].

**Table 1:** A list of various gut microbiota that affect angiogenesis

Microbe type	Gut microbe species	Angiogenic signaling pathways/growth factors/cell types	Affected organs/vascular beds	Probiotic application tested (if applicable)	Evidence type (association/causation)
Symbionts	Indigenous gut microbiota/ *B. thetaiotaomicron*	Paneth cells	Small intestine/intestinal villi	N/A	Causation (the formation of capillary networks is inhibited in GF mice and mice lacking Paneth cells) [16]
Symbionts	Indigenous gut microbiota	TF–PAR1 signalling loop/Angiopoietin-1 (Ang-1)/Endothelial Cells, Enterocytes	Small Intestine/Intestinal Villi	N/A	Causation (GF mice have impaired intestinal vascular remodeling, and TF-PAR1 is involved in intestinal vascular remodeling) [11]
Symbionts	Indigenous gut microbiota	TLR4 signaling/Endothelial Cells	Brain/cerebral cavernous malformations	N/A	Causation (*Gram-negative bacteria* or lipopolysaccharides (LPS) activate TLR4, accelerating CCM formation, which does not occur in GF mice) [20]
Symbionts	*Firmicutes* /*Bacteroidetes*	Inflammaging pathways/IL-6, IL-1β, TNF-α, VEGF-A/Endothelial Cells	Eye/choroidal neovascularization	N/A	Causation (high-fat diet alters gut microbiota, leading to increased intestinal permeability and chronic inflammation) [20]
Symbionts	Indigenous gut microbiota/ *Escherichia coli*	TLR4/TRIF signaling pathway/Neutrophils	Small intestine/mesenteric venules	N/A	Causation (the gut microbiome inhibits NETosis in mesenteric ischemia–reperfusion injury, whereas NETosis is enhanced in GF mice) [41]
Symbionts	*Escherichia coli 0111:B4 (LPS-EB*	TLR4/MYD88 signaling pathway/VEGF, PDGF-BB/Outgrowth Endothelial Cells (OECs), Primary Human Osteoblasts (pOBs)	Bone/osteoblast-endothelial interface	N/A	Causation (TLR4 activation by LPS-EB significantly enhances microvessel formation in co-culture systems) [42]
Symbionts	*Afa/Dr diffusely adhering E. coli*	HIF-1α signaling/IL-8, VEGF/Intestinal Epithelial Cells	Intestine/microvasculature	N/A	Causation [43]
Pathobionts	*Escherichia coli*	Not specified	Liver/colorectal cancer metastasis	N/A	Causation (Tumor-resident bacteria *E. coli* disrupt the GVB, leading to bacteria dissemination to the liver and promotion of PMN) [44]
Pathobionts	*Fusobacterium nucleatum*	VEGF receptor modulation/Human Umbilical Vein Endothelial Cells (HUVECs)	Vascular/endothelium	N/A	Causation (*F. nucleatum* impairs endothelial cell proliferation and tube formation) [45]
Probiotics	*Bacteroides fragilis ATCC25285*	TLR/NF-κB pathway inhibition/Colonic cells (hcoEPIC), Immune cells	Intestine/colon	Oral administration of live B. fragilis to mice for 21 days, followed by DSS-induced colitis model	Causation (B. fragilis ameliorates DSS-induced colitis, decreases inflammatory cytokines, and increases IL-10) [46]
Pathobionts	*Bacteroides fragilis*	VEGF/Colon cancer cells (B51LiM)	Colorectal tumor recurrence	N/A	Causation (Postoperative intra-abdominal infection increases VEGF levels, angiogenesis, and tumor recurrence) [47]
Pathobionts	*Bacteroides fragilis*	E-cadherin/β-catenin/NF-κB pathway/Intestinal epithelial cells (HT29/C1)	Not specified	N/A	Causation (BFT induces ectodomain cleavage of E-cadherin and IL-8 secretion through β-catenin, NF-κB, and MAPK pathways) [48]
Pathobionts	*Enterococcus faecalis*	VEGF/Retinal cells, Endothelial cells	Eye/retina	N/A	Association (Enterococcus colonization shows poor outcomes in anti-VEGF therapy for endophthalmitis) [49]
Pathobionts	*Enterococcus faecalis*/BV	PI3K/AKT/mTOR signaling pathway/IL-8、VEGFA/Colorectal cancer cells	Colorectal cancer	N/A	Causation (E. faecalis and its metabolite biliverdin promote colorectal cancer progression) [50]
Probiotics	*Enterococcus faecalis KH2*	TNF-α, IL-6, bFGF, (TGF)-β1, VEGF/Keratinocytes, Fibroblasts, Leukocytes	Skin/wound bed	Topical administration of heat-killed E. faecalis *KH2* strain	Causation (Heat-killed KH2 promotes re-epithelialization and granulation tissue formation) [51]
Pathobionts	*Enterococcus faecalis*	EMT/M2-like macrophage polarization/Keratinocytes, Fibroblasts, Macrophages, Neutrophils	Skin/wound bed	N/A	Causation(E. faecalis infection causes delayed wound healing) [52]
Symbionts	*Bacillus genus*/DMA	Acetylation of Cyclin A/VEGFB/Vascular endothelial cells, Skeletal muscle cells	Skeletal muscle/vascular system	N/A	Causation (d-malate inhibits skeletal muscle growth and angiogenesis via acetylation of Cyclin A) [18]
Probiotics	*Lactiplantibacillus plantarum UBLP-40/ Lactobacillus rhamnosus UBLR-58/ Bifidobacterium longum UBBL-64*	Pro-inflammatory, healing, and angiogenetic factors/Various skin cells including keratinocytes, fibroblasts	Skin/wound bed	Topical application in a rat excisional wound model	Causation (UBLP-40 aids wound healing, UBLR-58 enhances healing factor expression more effectively than UBBL-64, but UBBL-64 is stronger in promoting angiogenic factors) [53]
Probiotics	*Lactobacillus rhamnosus GG*	VEGF, AKT and HIF1α signaling pathways/VEGF, Bcl-2, EGFR/Gastric mucosal cells, Keratinocytes, Endothelial cells	Stomach/gastric ulcer	Intragastric administration at 10^8^ cfu/day or 10^9^ cfu/day for three consecutive days after ulcer induction	Causation (L. rhamnosus GG reduces gastric ulcer area, stimulates angiogenesis) [54]
Probiotics	*Lactobacillus rhamnosus SHA113*	Anti-oxidant and anti-inflammatory pathways/VEGF, TGF-β, EGF/Gastric epithelial cells (GEC-1), Macrophages (RAW264.7), Endothelial cells	Stomach/gastric ulcer	Oral administration of L. rhamnosus SHA113 cells and culture supernatant in a mouse model of alcoholic gastric ulcers	Causation (L. rhamnosus SHA113 and SHA-FS promote healing of alcoholic gastric ulcers) [55]
Probiotics	*Akkermansia muciniphila*	PDGF-BBs/Preosteoclasts, Endothelial cells	Bone/fracture site	Intragastric administration in fractured mice	Causation (A. muciniphila promotes type H vessel formation, thereby enhancing fracture healing) [56]
Probiotics	*Akkermansia muciniphila*	Inflammatory memory pathways, Angiogenic pathways/Keratinocytes, Fibroblasts, Endothelial cells	Skin/wound bed	Self-assembling nanofiber hydrogel scaffold seeded with A. muciniphila	Causation (A. muciniphila promotes angiogenesis and wound healing in diabetic ischemic ulcers) [57]
Probiotics	*Bacillus polyfermenticus*	NF-κB and IL-8 signaling pathways/IL-8/HIMECs	Intestine/microvasculature	Conditioned medium (CM) of B. polyfermenticus	Causation (B. polyfermenticus increases angiogenesis in HIMECs) [58]
Probiotics	Kefir water (contains a mixture of probiotics)	Antimetastatic and antiangiogenic pathways/breast cancer cells	Breast/tumor	N/A	Causation (Proinflammatory and proangiogenic markers were significantly reduced in the kefir water group) [59]
Probiotics	*Lactobacillus rhamnosus GG*	FPR1-mediated proresolving and antiangiogenic pathways/Colorectal carcinoma cells	Colon/colorectal carcinoma	LGG culture supernatant treatment	Causation (LGG activates FPR1, leading to suppression of angiogenic factors in CRC cells) [60]
Pathobionts	Clostridium perfringens type C/beta-toxin (CPB)	porcine endothelial cells	Intestine/microvasculature	N/A	Causation (CPB destroys intestinal microvasculature) [61]
Pathobionts	*Pseudomonas aeruginosa*/ azurin/ p28 peptide	VEGFR-2, FAK, and Akt pathways/VEGF, bFGF/HUVEC	Various/tumor	N/A	Causation (p28 peptide inhibits capillary tube formation, and neoangiogenesis) [62]
Pathobionts	*Bacillus anthracis*/ High-affinity anthrax toxin receptor (ATR) ligands	VEGF and bFGF signaling pathways/VEGF, bFGF/Endothelial cells, Tumor cells	Various/tumor	N/A	Causation (Mutant PA inhibits angiogenesis and tumor growth by disrupting receptor binding)
Pathobionts	*Bacillus anthracis/ anthrax lethal toxin (LeTx)*	MAPK signaling pathways/ VEGF, bFGF/Endothelial cells, Tumor cells	Various/tumor	N/A	Causation (MMP-activated LeTx inhibits angiogenesis and tumor growth by reducing endothelial cell migration and vascularization) [63]
Pathogens	*Helicobacter pylori*	NF-κB signaling pathways/ IL-8/HUVECs	Stomach/gastric mucosa	N/A	Causation(H. pylori colonization of the gastric mucosa promotes the expression of inflammatory factors, leading to chronic inflammation) [64]
Pathogens	*Helicobacter pylori*	MAPK/ERK1/2 signaling pathways/VEGF/Gastric epithelial cells, Endothelial cells	Stomach/gastric mucosa	N/A	Causation (PPIs suppress H. pylori-induced angiogenesis by inhibiting MAPK/ERK1/2 phosphorylation and downregulating VEGF expression) [65]
Pathogens	*Helicobacter pylori*	COX-2/Wnt/beta-catenin/VEGF pathway/VEGF/Gastric cancer cells, Endothelial cells	Stomach/gastric mucosa	N/A	Causation (H. pylori infection increases VEGF expression via the COX-2/Wnt/beta-catenin pathway, enhancing angiogenesis in gastric cancer) [66]

## The impact of gut microbiota and its metabolic products on pathological angiogenesis.

**Fig. 1:**
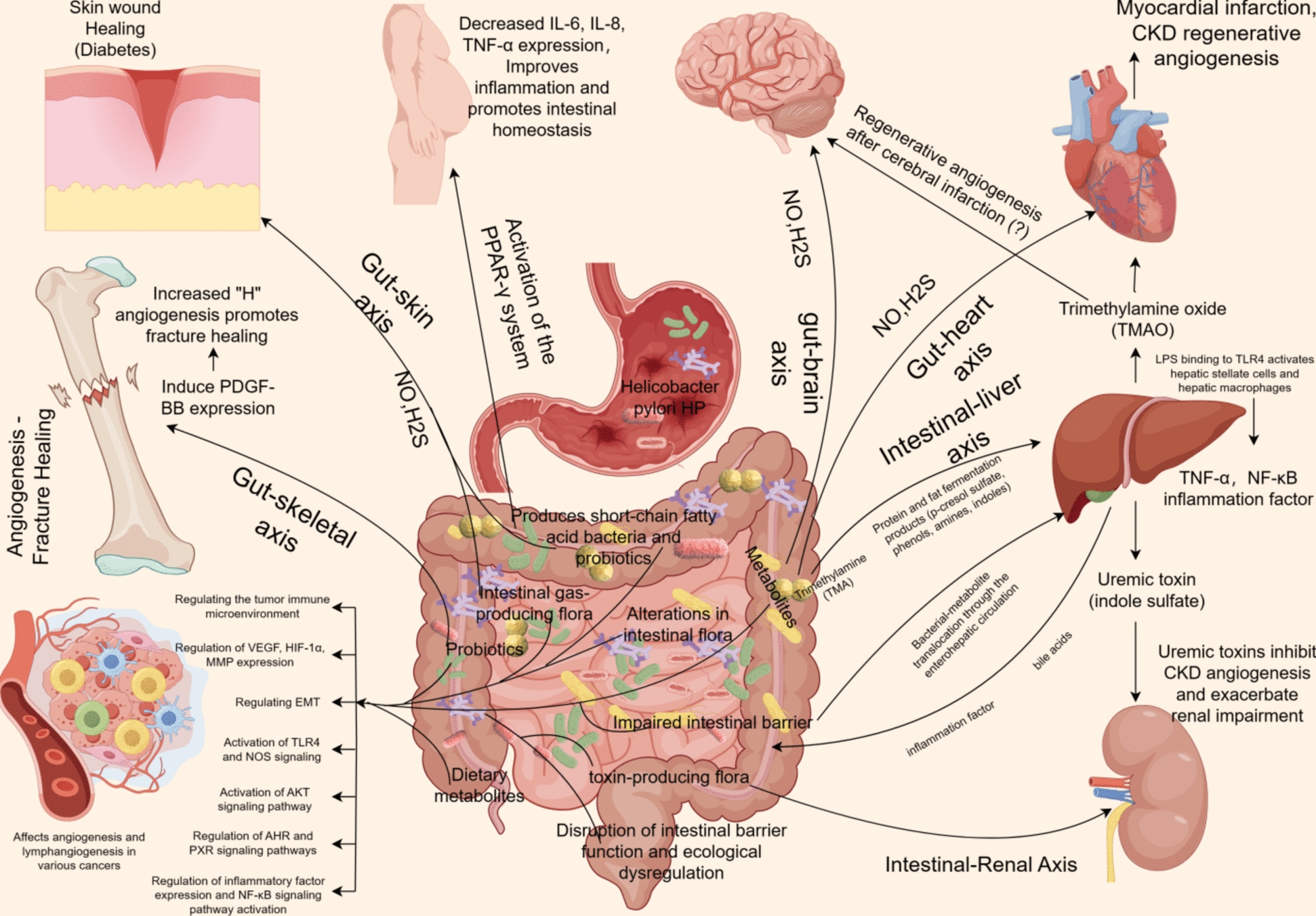
The intestinal flora and their metabolites form a complex network that significantly influences angiogenesis and lymphangiogenesis by fine-tuning key biosignaling axes in disease progression (e.g., brain-gut, gut-liver, gut-kidney, gut-skin, gut-heart, gut-skeletal, and gut-metabolism axes, among others), which in turn influences the regression of the disease

An increasing body of research substantiates the significant connection between the gut microbiota and distant organs(Fig. 1). *Gram-negative bacteria* or LPS in the gut accelerate the formation of cerebral cavernous malformations (CCMs) through the TLR4 pathway, a phenomenon that is not observed in germ-free (GF) mice and in normal mice treated with antibiotics [19]. Mice fed a high-fat diet exhibit changes in gut microbiota composition (an increase in the relative abundance of *Firmicutes* and a decrease in *Bacteroidetes*). This dysbiosis increases intestinal permeability and chronic low-grade inflammation, leading to pathological angiogenesis, such as age-related macular degeneration (AMD) [20]. Dysbiosis of the gut microbiome can compromise the integrity of the intestinal barrier, facilitating the translocation of bacteria or their metabolic byproducts into the bloodstream. This process may initiate immune metabolic responses in remote organs, culminating in chronic inflammation, the secretion of inflammatory mediators, and the upregulation of vascular endothelial growth factor (VEGF) expression. Such alterations can contribute to pathological angiogenesis, as observed in various retinal diseases [67]. Another example is the impact of the gut-lung axis on asthma. A Mendelian randomization study identified a potential causal relationship between gut microbiota and asthma. The class *Bacilli* and the order Lactobacillales were associated with a reduced risk of asthma, whereas eosinophilic asthma was linked to lower levels of indole propionic acid [68]. This suggests that the effects of gut microbiota metabolites on inflammatory responses may influence their role in asthma.The colonization of gut microbiota and its metabolic byproducts may exert an influence on angiogenesis in colorectal cancer (CRC) as well as in other distant organs, including gastric and liver cancers, through either direct contact or systemic circulation. This influence may either promote or inhibit angiogenesis, which could have significant implications for tumor metastasis. Furthermore, gut microbiota and its metabolic byproducts may contribute to tissue repair by enhancing inflammatory responses and angiogenesis, potentially affecting conditions such as fractures and wound healing. However, additional research is required to substantiate these claims.

The primary objective of this review is to elucidate the effects of gut microbiota and its metabolites on angiogenesis and lymphangiogenesis (Tables 1, 2). This synthesis aims to inform therapeutic strategies that can modify the composition of gut microbiota and enhance the production of its metabolites.

**Table 2:** A list of various gut microbiota metabolites that affect angiogenesis

Metabolite type	Metabolite	Angiogenesis signaling pathway/growth factor/cell type	Affected organ/vascular bed	Evidence type (association/causation)
Secondary bile acids	chenodeoxycholic acid (CDCA)	VEGFR, MMP9/HUVECs	Vascular system/zebrafish embryos	Causation (CDCA enhances ectopic angiogenesis in zebrafish embryos, increasing the expression of VEGFR and MMP9) [113]
	Lithocholic acid (LCA)	HUVECs	Vascular System/zebrafish embryos	Causation (LCA reduced the tubular structure formation of HUVECs and was toxic to zebrafish embryos) [113]
	Ursodeoxycholic Acid (UDCA)	VEGF-STAT3 signaling axis/VEGF/Retinal cells	Retina/ Oxygen-Induced Retinopathy(OIR)	Causation (UDCA prevents pathological neovascularization in the OIR mouse model) [114]
	Tauroursodeoxycholic Acid (TUDCA)	NF-κB, VEGF/VEGF/Human Retinal Microvascular Endothelial Cells (HRMECs)	Retina/diabetic retinopathy(DR)	Causation (TUDCA inhibits pathological neovascularization in a rat model of DR) [115]
	Lithocholic Acid (LCA)	Erk1/2 MAPK and suppression of STAT3 activity/IL-8/Human colorectal cancer cells	Colon/Tumor blood vessels	Causation(LCA induces tumor cells to produce IL-8, promoting endothelial cell proliferation and tube formation) [126]
	Lithocholic Acid (LCA)	TGR5 receptor/VEGF/Breast Cancer Cells	Breast/Tumor blood vessels	Causation (LCA inhibits tumor cell proliferation, VEGF production, and EMT) [127]
Gut Microbiota Metabolite	Trimethylamine N-oxide (TMAO)	PKC/NF-κB/VCAM-1 signaling pathway/HUVECs	Endothelium, Arterial system	Causation (TMAO impairs endothelial self-repair capacity and enhances monocyte adhesion) [131]
	Trimethylamine N-oxide (TMAO)	Purinergic signaling pathway/Bovine aortic endothelial cells (BAE-1)	Endothelium	Causation (TMAO induces the release of NO from endothelial cells and the phosphorylation of eNOS, affecting vascular dilation) [144]
	Trimethylamine N-oxide (TMAO)	VEGF-STAT3 signaling axis, PI3K/AKT/mTOR pathway/Colorectal cancer cells	Colon/Tumor blood vessels	Causation (TMAO promotes cell proliferation and angiogenesis in colorectal cancer) [150]
	Trimethylamine N-oxide (TMAO)	MAPK/ERK and NF-κB signaling pathways/Endothelial cells (HAECs), Vascular smooth muscle cells (VSMCs)	Vascular system/aortas	Causation (TMAO Promotes Vascular Inflammation and Atherosclerosis) [135]
	Trimethylamine N-oxide (TMAO)	CXCR4 signaling/ Hepatocytes, Vascular endothelial cells (VECs)	Liver, Vascular system	Causation (TMAO inhibits the expression of CXCR4, impairs the migration and tube formation of VECs, and hinders the recovery of ischemic reperfusion and angiogenesis in the hind limbs of mice) [136]
Short-Chain Fatty Acids(SCFAs)	Butyrate	NF-κB and PPARα signaling pathways/HUVECs	Endothelium	Causation (Butyrate inhibits the activation of NF-κB and PPARα in endothelial cells, exerting anti-inflammatory effects) [193]
	Butyrate	PPAR-γ signaling/Colonocytes, Immune cells	Colon	Causation (Butyrate inhibits dysbiosis of potentially pathogenic Escherichia coli and Salmonella through PPAR-γ signaling) [151]
	Acetate, Butyrate, Propionate	GPR41/43 signaling, HDAC inhibition/HUVECs	Endothelium	Causation (SCFAs activate GPR41/43 in HUVECs and inhibit HDACs to exert anti-inflammatory and anti-adhesive effects) [154]
	Butyrate	HIF-1α signaling pathway/VEGF/Intestinal tumor cells, Endothelial cells	Intestine/tumer vascular system	Causation (Butyric acid inhibits angiogenesis in intestinal cancer cells by downregulating HIF-1α and VEGF) [157]
	Butyrate	Sp1 transactivation/NRP-1, VEGF/Colorectal cancer cell lines (Caco-2, HCT116, HT29)	Colon/tumer vascular system	Causation (Butyrate suppresses NRP-1 expression and Inhibits Angiogenesis) [160]
Gut Microbiota Metabolite	Lysophosphatidic acid (LPA)	YAP/TAZ activation, Notch signaling/Notch ligand Dll4/Endothelial cells	Vascular system, particularly during development	Causation (LPA promotes developmental angiogenesis by repressing Dll4) [168]
	LPA	LPA2 signaling through PI3K-Akt/PLC-Raf1-Erk pathways/Endothelial cells	Heart, Vasculature	Causation (LPA-LPA2 signaling maintains vascular endothelial homeostasis and plays a protective role in a mouse model of myocardial infarction (MI)) [170]
	LPA	Gut Microbiota Modulation	Colon,tumer vascular system	Causation(HFD mouse fecal microbiota transplantation (FMT) promotes the occurrence of colon tumors in AOM-treated germ-free (GF) mice) [171]
	LPA	LPA4 signaling/Endothelial cells	tumer vascular system	Causation (LPA-induced normalization of tumor blood vessels helps in tumor drug therapy) [175]
Indole derivatives	Indole-3-aldehyde (IAld), Indole-3-acetic Acid (I3AA)	AHR, NF-κB, MAPK pathways/IL-1β, IL-6, VEGFA/Murine RAW 264.7 (RAW) cells, Endothelial cells	Joints, Vasculature	Causation (IAld and I3AA have different effects on the expression of VEGF in endothelial cells) [178]
	Indoxyl sulfate (IS)	NADPH oxidase (NOX), Protein Kinase C (PKC), eNOS phosphorylation/HAECs	Vasculature, particularly in chronic kidney disease(CKD)	Causation (IS inhibits valsartan-induced neovascularization in CKD) [181]
	Indole 3-propionic acid (IPA)	PXR signaling pathway/NO/Endothelial cells	Vasculature	Causation (IPA regulates endothelial cell function by affecting PXR) [182]
Polyphenol	(-)-Epigallocatechin-3-gallate (Pro-EGCG)	PI3K/AKT/mTOR/HIF1α pathway/VEGFA/Endometrial cancer cells, Tumor-associated macrophages (TAMs)	Endometrium	Causation (Pro-EGCG inhibits angiogenesis in endometrial cancer) [221]
	Theaflavin-3,3’-digallate (TF3)	Akt and Notch-1 pathways/VEGF, HIF-1α/Human ovarian carcinoma OVCAR-3 cells,HUVECs	Ovary, Vasculature	Causation (TF3 inhibits OVCAR-3 cell-induced angiogenesis) [222]
Gut microbiota-produced gas	Hydrogen Sulfide (H2S)	NOS/NO pathway/ Neural cells, Endothelial cells	Brain vasculature in zebrafish	Association (H2S promotes developmental brain angiogenesis) [189]
	H2S	NOS/NO pathway/VEGF/Endothelial cells	Hind limb vasculature in rats	Association (H2S promotes angiogenesis in hind limb ischemia) [190]
	H2S	AKT/eNOS signaling, Angiopoietin-1/VEGF, Ang-1/Endothelial progenitor cells	Skin, Vasculature in type 2 diabetes	Association (H2S promotes wound healing) [191]
	H2S	AKT/eNOS, VEGF/NO, JAK2/STAT3 pathways/VEGF, Ang-1/Cardiac cells, Endothelial cells	Heart, Coronary vasculature	Association (H2S plays a protective role in acute myocardial infarction and heart failure) [192]
	H2S	PI3K/Akt pathway/VEGF-C/Lymphatic endothelial cells (LECs)	Lymphatic system	Association (H2S promotes lymphangiogenesis and alleviates lymphedema) [196]
	H2S	HIF-1α activation, PI3K/Akt pathway/VEGF/Non-small cell lung cancer cells (NSCLCs), Endothelial cells	Lung, Vasculature	Association(H2S promotes angiogenesis in NSCLC) [197]
	H2S	HIF-1α, AP-1, VEGF/Colon cancer cells	Colon,tumer vascular system	Association(H2S promotes liver metastasis of colon cancer by upregulating VEGF through AP-1 activation) [198]
	H2S	STAT3 pathway/VEGF, HIF-1α/Hepatocellular carcinoma cells (HepG2, Bel7402)	Liver	Association(H2S shows effective anti-hepatocellular carcinoma activity through blocking the STAT3 pathway) [203]
	H2S	EGFR/ERK/MMP-2, PTEN/AKT pathways/Hepatocellular carcinoma cells	Liver	Association(H2S exerts a dual role in HCC cells through the EGFR/ERK/MMP-2 and PTEN/AKT signaling pathways) [204]
	Nitric Oxide (NO)	VEGF-C signaling pathway/VEGF-C/Breast cancer cells	Breast tissue, Lymphatic system	Association(NO promots VEGF-C expression and lymphangiogenesis in breast cancer) [211]
	NO	cGMP-PKG-ERK signaling pathway/MMP-2/9/Colon cancer cells	Colon	Association (NO promots migration/invasion by upregulating MMP-2/9) [212]
	NO	NO-mediated pathways/Tumor endothelial cells	Tumor vasculature	Association (NO promots tumor vessel normalization) [214]

### The impact of gut microbiota on pathological angiogenesis

#### Escherichia coli

Lipopolysaccharide (LPS) is a constituent of the outer membrane of *Gram-negative bacterial* cell walls. Under physiological conditions, LPS is typically retained within the bacterial structure; however, it is released upon the death and subsequent lysis of the bacterial cell [69]. Research indicates that LPS can upregulate the expression of DDL4 and enhance angiogenesis in human pulmonary microvascular endothelial cells (HPMECs) through the activation of the Toll-like receptor 4 (TLR4)-extracellular signal-regulated kinase (ERK)-forkhead box C2 (FOXC2) signaling pathway. Furthermore, systemic administration of LPS has demonstrated a significant impact on retinal angiogenesis in murine models [70]. LPS derived from *Escherichia coli* promote angiogenesis and osteogenesis via the TLR4-MYD88 signaling pathway [42].

**Fig. 2:**
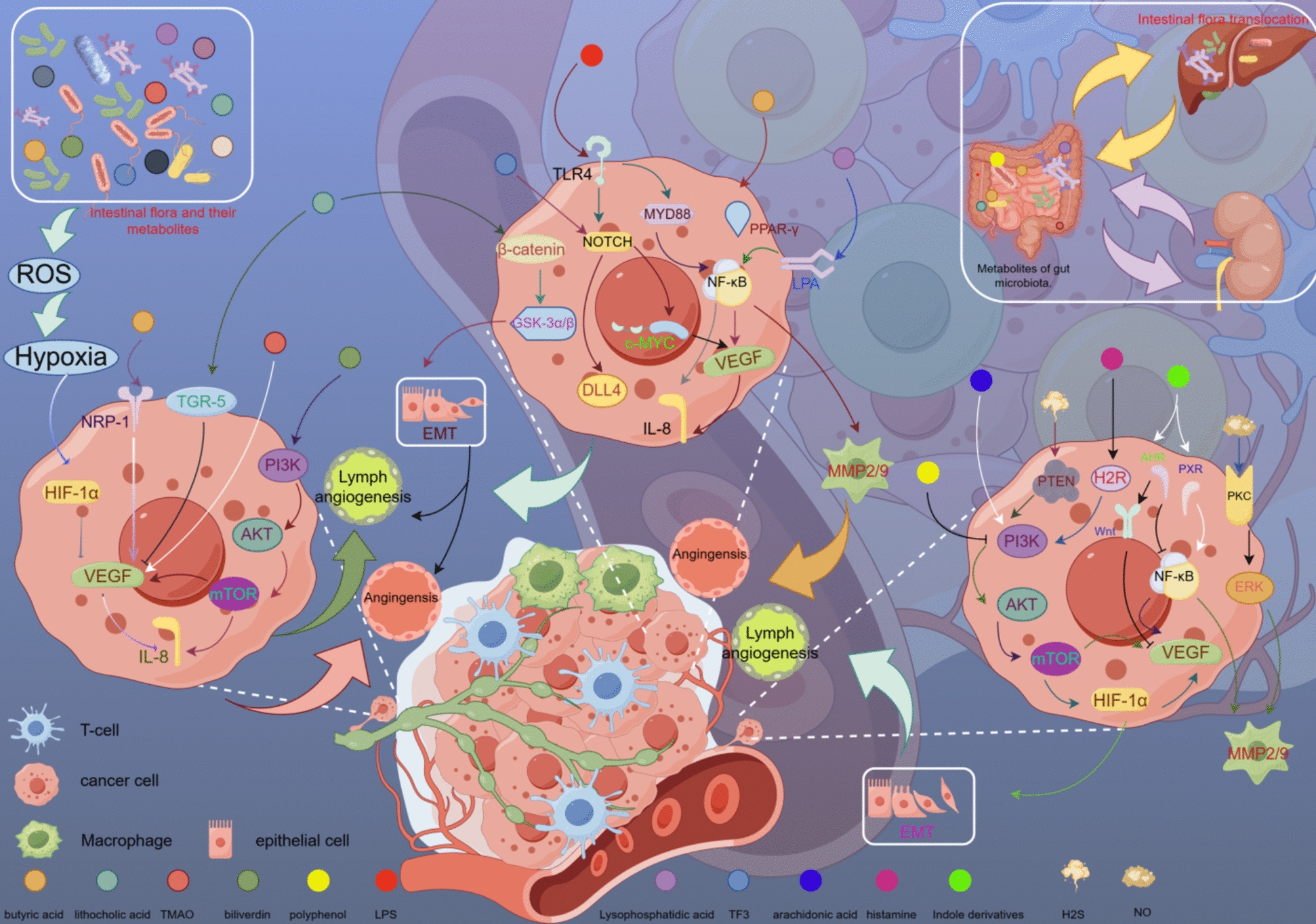
The gut flora and its metabolites exert a critical influence by acting on various signaling pathways within tumor cells. These include modulation of inflammatory processes, interference with tumor immune responses, influence on epithelial-mesenchymal transition (EMT), PI3K/AKT signaling pathways, activation of Toll-like receptors (TLRs), signaling through aromatic hydrocarbon receptor (AHR) and pregnane X receptor (PXR), and lysophosphatidic acid (LPA)-mediated signaling pathways. These actions further regulate the secretion of pro-angiogenic and pro-lymphangiogenic factors by tumor cells, thereby affecting the formation of vascular and lymphatic networks in tumor tissues. These complex biological mechanisms have profound implications for tumor growth, metastasis and survival

In colorectal cancer (CRC) and ulcerative colitis (UC), the afa-1 operon in *E. coli* has been demonstrated to induce the overexpression of HIF-1α and upregulate the expression of IL-8, VEGF, and Twist1 genes, while simultaneously downregulating the expression of E-cadherin (Fig. 2). This process, known as epithelial-mesenchymal transition (EMT), facilitates the formation of new blood and lymphatic vessels in CRC and UC [43, 71]. During the progression of CRC, *E. coli* can disrupt the gut vascular barrier (GVB), thereby initiating the formation of pre-metastatic niches (PMN) [44]. This disruption triggers inflammatory responses and the formation of blood and lymphatic vessels, thus promoting the dissemination and metastasis of cancer cells [44, 72–76]. In CRC patients, elevated PV-1 levels (a GVB injury marker) were linked to liver bacterial dissemination and asynchronous distant metastasis, suggesting its potential as a prognostic marker for distant recurrence and liver metastasis from vascular injury [44].

#### Fusobacterium nucleatum

*Fusobacterium nucleatum* has been shown to enhance the proliferation of endothelial cells and promote angiogenesis by upregulating vascular endothelial growth factor (VEGF) and its receptor (VEGFR) in human umbilical vein endothelial cells (HUVECs). Additionally, it facilitates the release of prostaglandin E2 (PGE2), which is involved in vasodilation. This pro-angiogenic effect may be associated with the inflammatory changes in vascular endothelial cells induced by stimulation from *F. nucleatum* [45].

In CRC, *F. nucleatum* activates the TLR4-MYD88-NF-κB signaling pathway, leading to the upregulation of miR21 and the downregulation of RAS GTPase (RASA1) [77]. This results in the sustained activation of the RAS-MAPK pathway [78, 79], thereby promoting the formation of blood and lymphatic vessels in CRC [80–82]. Moreover, the activation of the TLR4-MYD88-NF-κB signaling pathway intensifies inflammatory responses by elevating the expression of inflammatory mediators, including COX-2, IL-8, TNF-α, IL-6, and IL-1β [83, 84]. This upregulation results in increased expression of VEGF, which in turn promotes angiogenesis and lymphangiogenesis.

#### Bacteroides fragilis

*Bacteroides fragilis* is categorized into enterotoxin-producing (ETBF) and non-enterotoxin-producing (NTBF) strains. NTBF shows probiotic potential by downregulating the NF-κB pathway, reducing pro-inflammatory factors IL-6 and IL-1β, and increasing the anti-inflammatory factor IL-10. This alleviates TNF-α-induced inflammation, as demonstrated in a DSS-induced mouse colitis model, and may indirectly affect angiogenesis [46]. The weakly virulent *Bacteroides fragilis* toxin (BFT), secreted by enterotoxigenic *Bacteroides fragilis* (ETBF), induces the degradation of the E-cadherin protein in intestinal epithelial cells. This process activates the β-catenin-TCF nuclear signaling pathway [48], which in turn stimulates the production of interleukin-8 (IL-8) [85], thereby promoting inflammatory responses and angiogenesis.

Through the establishment of a postoperative abdominal infection model in mice via intraperitoneal injection of *Bacteroides fragilis*, researchers have demonstrated that abdominal infections induced by *Bacteroides fragilis* may facilitate tumor recurrence by enhancing angiogenesis [47]. The nuclear localization of β-catenin induced by BFT can enhance the expression of the oncogene c-Myc [86], consequently facilitating tumor angiogenesis and lymphangiogenesis through multiple signaling pathways, including the FGF-MYC-HK2 axis [87] and pathways associated with VEGF [88, 89]. It is important to note that *Bacteroides fragilis* may also provide a protective effect in the context of CRC. Xihuang Wan enhances the anti-cancer efficacy of Anlotinib by modulating the gut microbiota, such as NTBF, and by inhibiting tumor angiogenesis [90]. Research indicates that polysaccharide A (PSA) produced by *Bacteroides fragilis* modulates the cell cycle in CRC via a TLR2-mediated signaling pathway [91, 92].

#### Enterococcus faecalis

In a study on anti-VEGF-related endophthalmitis, cultures from the deteriorated vision group after intravitreal injection of anti-VEGF demonstrated a higher frequency of growth of *Enterococcus faecalis* [49].

In CRC, bilirubin (BV) has been demonstrated to significantly increase the expression levels of IL-8 and VEGF-A by regulating the phosphatidylinositol 3-kinase/Akt/mammalian target of rapamycin (PI3K/AKT/mTOR) signaling pathway, thereby promoting angiogenesis and lymphangiogenesis [50]. In chronic skin wounds, the application of *heat-killed Enterococcus faecalis* (*KH2*) has been demonstrated to promote re-epithelialization and the formation of blood vessels and granulation tissue. This is achieved by inducing the production of TNF-α, IL-6, basic fibroblast growth factor (bFGF), transforming growth factor (TGF)-β1, and VEGF, which facilitate wound healing [51]. This mechanism may prove beneficial in the treatment of chronic skin injuries in patients with diabetes. However, studies have shown that *Enterococcus faecalis* infection may cause delayed wound healing. *Enterococcus faecalis* infection leads to dysregulation of cellular behavior, promoting keratinocytes to undergo the epithelial-mesenchymal transition (EMT) process prematurely and incompletely, and modulating the polarization of M2-like macrophages, which induces pathological angiogenesis and results in delayed wound healing [52].

#### Gut probiotics

Probiotics play a pivotal role in maintaining the ecological balance of the gut. Probiotics regulate intestinal mucosal inflammation by activating VEGFR signaling and stimulate angiogenesis, which helps repair damaged intestinal mucosa and promote gut health [93]. Probiotics improve lipid metabolism, glucose metabolism, and cholesterol metabolism, inhibit oxidative stress, and reduce the production of chronic inflammation, which aids in the treatment and prevention of metabolic diseases such as obesity, diabetes, and cardiovascular diseases [94]. d-malic acid (DMA) derived from the *Bacillus genus* has been shown to induce impaired angiogenesis in skeletal muscle through the acetylation of cyclin A during the aging process, ultimately leading to skeletal muscle atrophy. This may provide new insights into the skeletal muscle atrophy in the elderly stemming from the gut microbiota [18].

Probiotics have been shown to play a beneficial role in the process of wound healing. *Lactobacillus rhamnosus GG (LGG)* and *Bifidobacterium longum* have been observed to enhance wound healing and angiogenesis in rat skin excision models. Notably, *Bifidobacterium longum* exerts a more pronounced effect on the expression of pro-angiogenic factors, whereas *LGG* is more effective in promoting the expression of healing factors [53]. The mechanism by which probiotics facilitate wound healing includes the exertion of anti-inflammatory effects; for instance, *LGG* demonstrates both anti-inflammatory and pro-angiogenic properties that contribute to the healing of gastric ulcers [54, 55]. Furthermore, *Akkermansia muciniphila* administered intraperitoneally alleviates post-fracture inflammation by inhibiting the release of pro-inflammatory mediators, while also enhances the secretion of platelet-derived growth factor-BB (PDGF-BB) from pre-osteoblasts, thereby stimulating the formation of H-type blood vessels and promoting fracture healing [56, 95–99]. Additionally, the combination of *Akkermansia* with functional peptide gel, followed by subcutaneous administration, has been shown to facilitate the healing of diabetic ischemic ulcers [57]. *Bacillus polyfermenticus (B.P)* enhances the migration, permeability, and tubular structure formation of human intestinal microvascular endothelial cells (HIMECs) through the NF-κB/IL-8/CXCR2 signaling pathway (Fig. 2), promoting angiogenesis in HIMECs. In a mouse intestinal inflammation model, it facilitates intestinal repair and healing [58].

Probiotics play a key role in preventing tumor metastasis [100]. Probiotics can increase the adhesion of tumor cells by regulating the levels of tight junction proteins ZO-1 and E-cadherin [101–103], while also inhibiting tumor EMT and the expression levels of VEGF and MMPs [104, 105], thereby suppressing the degradation of the tumor basement membrane and inhibiting angiogenesis.Probiotic treatment reduced the expression of EMT-related markers (Snail-1 and ZEB-1) in a mouse model of pancreatic cancer [104]. Probiotic fermented food kefir exhibited anti-metastatic and anti-angiogenic effects in mouse breast cancer cells, leading to the upregulation of tissue inhibitors of metalloproteinases (TIMPs) [59]. *LGG* promotes the resolution of inflammatory responses in the tumor microenvironment and inhibits tumor angiogenesis by activating formyl peptide receptor 1 (FPR1) [60].*Akkermansia muciniphila* induce an anti-tumor immune response and exert a protective effect by increasing CD8 + cytotoxic T lymphocytes (CTL) in a mouse model of colitis-associated colorectal cancer (CAC) [106, 107]. *Lactobacillus acidophilus* and *Lactobacillus plantarum* produce extracellular polysaccharides (EPS), which may play a role in inhibiting tumor angiogenesis [108]. EPS exerts its anti-inflammatory effects by downregulating the expression of VEGF in tumor cells, upregulating the expression of the anti-angiogenic factor tissue inhibitor of metalloproteinases-3 (TIMP-3) [109], downregulating pro-inflammatory factors, and upregulating anti-inflammatory factors [110]. In a mouse melanoma model, EPS inhibited tumor angiogenesis [111].

### The impact of gut microbiota-derived metabolites on the development of vascular and lymphatic vessels

#### Secondary bile acids

Secondary bile acids are recognized as potent signaling molecules that can modulate various receptors influencing metabolism and lipid distribution, including farnesoid X receptor (FXR) and G protein-coupled bile acid receptor 1 (TGR5). The activation of these nuclear receptors may play a crucial role in establishing connections between the gut microbiota and cardiovascular health [112]. Research has demonstrated that hydrophobic bile acids, such as lithocholic acid (LCA) and deoxycholic acid (DCA), as well as hydrophilic bile acids, including cholic acid (CA) and chenodeoxycholic acid (CDCA), exert distinct effects on angiogenesis. Specifically, hydrophilic CDCA promotes the formation of ectopic blood vessels in zebrafish embryos by upregulating the expression of VEGF and matrix metalloproteinase 9 (MMP9), while concurrently downregulating the expression of the adhesion protein vascular endothelial cadherin (VE-cadherin). Conversely, hydrophobic LCA has been shown to inhibit nephron formation and exhibit toxicity towards zebrafish embryos [113]. Ursodeoxycholic acid (UDCA) has been demonstrated to inhibit pathological neovascularization in the retina by downregulating the expression of inflammatory factors and normalizing the VEGF-STAT3 signaling axis, which aids in the treatment of retinopathy of prematurity (ROP) and diabetic retinopathy [114–116]. UDCA downregulates the phosphorylation of NF-κB and STAT3 by negatively regulating the expression of suppressor of cytokine signaling 1 (SOCS1) and suppressor of cytokine signaling 3 (SOCS3). These alterations are associated with a decrease in angiogenesis, as evidenced by the downregulation of VEGF, vascular cell adhesion molecule (VCAM), and transforming growth factor-beta receptor II (TGF-βRII) expression. This suggests a potential therapeutic role for UDCA in the management of obesity and related metabolic disorders [117].

In recent years, the significance of secondary bile acids in the formation of blood and lymphatic vessels has attracted considerable interest from the scientific community [118–123]. Feces from patients with colorectal cancer exhibit elevated levels of secondary bile acids, as well as an increase in the gut microbiota that metabolizes these acids [124, 125]. Research has demonstrated that LCA can promote the metastasis of colorectal cancer. One study indicated that LCA activates the ERK1/2 signaling pathway and increases IL-8 levels by interacting with TGR5 on the surface of colorectal cancer cells. Furthermore, the supernatant from these cells can stimulate endothelial cell proliferation and tube formation [126]. However, a recent study indicates that physiological concentrations of LCA (< 1 μM) act on the surface TGR5 of tumor cells in both tumor-bearing mice and in vitro experiments. This action inhibits the proliferation of breast cancer cells and downregulates VEGF expression, thereby suppressing metastasis [127]. The concentration of LCA in the experiment (< 1 μM vs. 30 μM) may be significant. Lower LCA levels in normal tissues lead to fewer acute toxic effects, potentially enabling effective reduction of tumor cell proliferation and metastasis. The combination of low molecular weight heparin and deoxycholic acid (LHbisD4) has demonstrated efficacy in reducing tumor lymphatic metastasis by inhibiting VEGF-C induced lymphangiogenesis, and it exhibits low toxicity and oral bioactivity [128].

#### Trimethylamine N-oxide (TMAO)

TMAO induces inflammatory responses in endothelial cells and facilitate endothelial-mesenchymal transition (EndMT) [129]. Additionally, TMAO increases platelet reactivity and activates both platelet deposition and activation [130], thereby influencing the progression of atherosclerosis. TMAO also induces endothelial cell dysfunction and promotes apoptosis in vascular endothelial cells by increasing the expression of adhesion molecules [131]. In mouse models, the supplementation of TMAO can enhance cholesterol accumulation in macrophages and promote the formation of atherosclerotic plaques. In primary human aortic endothelial cells (HAECs) and vascular smooth muscle cells (VSMCs), TMAO activates the p38 mitogen-activated protein kinase (MAPK)/extracellular signal-regulated kinase (ERK)/nuclear factor kappa-light-chain-enhancer of activated B cells (NF-κB) signaling cascade, which induces the expression of inflammatory factors and may increase the risk of atherosclerosis and cardiovascular diseases [132–135]. These mechanisms are associated with adverse cardiovascular events and impede the ischemia–reperfusion process [136–143]. Furthermore, TMAO has been demonstrated to impede eNOS phosphorylation, consequently reducing nitric oxide release and inducing endothelial dysfunction. This, in turn, results in the inhibition of vascular relaxation and formation [144].

Recent studies have demonstrated that trimethylamine N-oxide (TMAO) can impede the formation of neutrophil extracellular traps (NETs) in the peripheral and umbilical cord blood plasma of women with gestational diabetes mellitus [145]. The inhibition of NETs has been observed to reduce their suppressive effect on vascular formation, thereby promoting fetal development. In colorectal cancer (CRC), TMAO has been identified as a contributing factor to tumor progression, particularly through the promotion of angiogenesis and lymphangiogenesis [146–149]. Through in vitro experiments, it was found that TMAO can induce the proliferation of CRC cells and upregulate the expression of VEGFA. At a concentration of 10 μmol/L, TMAO significantly promotes the proliferation of CRC cells. In in vivo experiments, elevated circulating levels of TMAO in tumor-bearing mice lead to a significant increase in tumor volume, new blood vessel formation, and the quantities of VEGFA and CD31 [150]. The tumor-promoting effects of TMAO help elucidate the correlation between high consumption of red meat and elevated choline intake with an increased risk of CRC.

TMAO can be seen as a new potential therapeutic target in vascular inflammation. By inhibiting the production of TMAO (through diet, probiotics, and targeted drugs), it may provide new treatment strategies for patients with cardiovascular diseases.

#### Short-chain fatty acids (SCFAs)

SCFAs are recognized for their crucial role in maintaining intestinal homeostasis. Research has demonstrated that antibiotic treatment in mice, which diminishes butyrate production by gut microbiota, leads to a reduction in the activation of the PPAR-γ signaling pathway. The absence of PPAR-γ signaling results in elevated nitrate levels and increased oxygen bioavailability in the intestine, thereby promoting the proliferation of potential pathogens such as *Escherichia coli* and *Salmonella* [151]. According to reports, short-chain fatty acids are closely related to the activation of the NLRP3 inflammasome and the process of angiogenesis, and they exhibit a protective effect on intestinal barrier function by inhibiting the NLRP3 inflammasome and autophagy [152]. In another study, butyrate inhibits the activation of NF-κB in human umbilical vein endothelial cells (HUVECs) and enhances the peroxisome proliferator-activated receptor (PPAR) signaling pathway, butyrate inhibits the expression of vascular cell adhesion molecule 1 (VCAM-1) and intercellular adhesion molecule 1 (ICAM-1) mRNA in HUVECs induced by cytokines such as TNF-α secreted by monocytes and lymphocytes [153]. Research has confirmed that the anti-inflammatory and anti-adhesion effects are mediated by the activation of G protein-coupled receptors 41 and 43 (GPR41 and GPR43) and the inhibition of histone deacetylases (HDACs). The activation of GPR41 and GPR43 inhibits the production of inflammatory factors, while the inhibition of HDACs downregulates the expression of VCAM-1 and reduces the adhesion of peripheral blood mononuclear cells (PBMCs) to the endothelial monolayer [154]. SCFAs may play a role in developmental angiogenesis and lymphangiogenesis. Research indicates that a deficiency in carnitine palmitoyltransferase 1A (CPT1A), a crucial rate-limiting enzyme in fatty acid β-oxidation, hinders the proliferation of endothelial cells. This impairment results in abnormalities in the development of both blood and lymphatic vessels. Acetic acid can be metabolized in the body to generate acetyl-CoA, which promotes fatty acid β-oxidation and mitigates the developmental defects in blood and lymphatic vessels associated with CPT1A deficiency [155, 156].

In cancer research, SCFAs are considered potential anti-angiogenic agents because they can influence the expression of VEGF.In colorectal cancer (CRC), sodium butyrate acts protectively by inhibiting VEGF and its key transcriptional activator, hypoxia-inducible factor (HIF)−1α [157, 158], thereby dampening hypoxia-induced angiogenesis [159]. Research has demonstrated that butyrate downregulates the expression of NRP-1 in CRC cells by inhibiting the transcriptional activation of Sp1 [160]. NRP-1 is a non-tyrosine kinase receptor that binds to specific members of the VEGF family [161] (VEGFRs are tyrosine kinase receptors [162]). Butyrate diminishes the interaction between NRP-1 and VEGF, thereby inhibiting tumor angiogenesis and tumor cell survival [163, 164]. In oral cancer, sodium butyrate significantly lowers VEGF-C expression, inhibiting lymphatic vessel formation [165].Butyrate's multifaceted influence points to its role in mitigating cancer progression through the suppression of tumor angiogenesis and lymphangiogenesis, highlighting the preventative potential of dietary interventions. Interestingly, butyrate can also promote angiogenesis at low doses by upregulating VEGF expression through the metabolite-sensing receptor GPR43, thus fostering angiogenesis and granulation tissue remodeling [153].

#### Lysophosphatidic acid (LPA)

The composition and metabolic activity of the gut microbiota can influence the levels of LPA. For instance, specific gut bacteria, including Lactobacillus and Bifidobacterium, are capable of producing phospholipase A2 (PLA2), which subsequently enhances the production of LPA. Furthermore, dysbiosis of the gut microbiota, commonly observed in various disease states, may result in alterations in LPA levels, potentially impacting the health of the host [166]. LPA plays a key role in embryonic angiogenesis [167–169]. LPA4/LPA6 double knockout mice exhibit embryonic lethality, while LPA6 knockout mice show impaired retinal angiogenesis. LPA activates YAP/TAZ through the LPA4 and LPA6 receptors, thereby inhibiting Dll4 expression and promoting developmental angiogenesis in mice [168]. The endothelial LPA-LPA2 signaling pathway promotes endothelial cell proliferation and angiogenesis, and maintains vascular homeostasis, which is crucial for restoring blood flow and repairing tissue function in ischemic injury [170].

Research has shown that fecal transplants from mice on a high-fat diet (HFD) to azoxymethane (AOM)-treated GF mice resulted in dysbiosis, metabolic disorders—particularly elevated LPA levels, and impaired intestinal barrier function in the GF mice, which promoted the development of colorectal tumors [171]. On one hand, LPA can induce the activation of the NF-κB pathway, leading to the release of the pro-inflammatory factor IL-8, which promotes tumor angiogenesis and lymphangiogenesis [172–174]. On the other hand, LPA induces the formation of submembranous circular actin bundles in endothelial cells through the activation of LPA4, and enhances the formation of VE-cadherin in linear adhesion junctions, which induces tumor vascular normalization and aids in tumor treatment [175].

#### Indole derivatives

In the gut, indole derivatives primarily act as agonists of the aryl hydrocarbon receptor (AHR) [176]. Indole derivatives activate the AHR signaling pathway in endothelial cells, thereby affecting the expression of VEGF and regulating the inflammatory response. For example, in rheumatoid arthritis, indole-3-aldehyde (IAld) stimulates angiogenesis by upregulating VEGF expression in endothelial cells, while indole-3-acetic acid (I3AA) downregulates VEGF expression to inhibit angiogenesis. The AHR antagonist CH-223191 is ineffective in suppressing the angiogenic effects of IAld but effectively reverses the anti-angiogenic effects induced by I3AA, indicating that the VEGF inhibitory effect of I3AA is dependent on the AHR pathway [177, 178]. Furthermore, activation of the AHR pathway promotes the production of the inflammatory mediator IL-22 in the intestinal mucosa, leading to increased blood pressure and endothelial dysfunction [179]. After activating the AHR pathway, indole sulfate inhibits cardiovascular system development by suppressing the Wnt signaling pathway [180]. In studies related to chronic kidney disease (CKD), it has been observed that indole sulfate reduces the vascular protective effects of RAAS inhibitors in CKD patients by inhibiting the NOX/PKC/eNOS pathway [181]. Indole-3-propionic acid (IPA), in addition to activating the AHR signaling pathway, also exerts anti-inflammatory effects through the PXR signaling pathway, reducing endothelial-dependent vascular reactivity inflammation [182, 183]. PXR-deficient mice demonstrate compromised intestinal barrier function, while PXR and TLR4 double knockout mice exhibit restored intestinal barrier integrity. This suggests that PXR activation safeguards intestinal barrier function by inhibiting TLR4 activation [184].

In a study investigating the relationship between diet and CRC, researchers found that, compared to the AOM/DSS + rice group of mice, the AOM/DSS + millet group altered the production of gut microbiota metabolites, including indole derivatives and short-chain fatty acids. This alteration activated the intestinal AHR and GPCRs signaling pathways while inhibiting STAT3 phosphorylation, which subsequently suppressed tumor cell proliferation and angiogenesis [185]. Indole derivatives may enhance the sensitivity of tumors to chemotherapy. For instance, Indole-3-Carbinol (I3C) inhibits the expression of NF-κB in tumor cells and cardiac tissue in a mouse model of breast cancer. It also downregulates the levels of VEGF-A and MMP-9 in serum, suppresses tumor angiogenesis, and increases the sensitivity of mouse breast cancer cells to doxorubicin (DOX) [186]. The aforementioned IPA can activate the PXR signaling pathway. Studies have demonstrated that rifampicin inhibits the release of pro-angiogenic mediators in colon cancer cells by activating PXR. This indicates that the PXR signaling pathway activated by IPA may influence tumor angiogenesis; however, relevant research in this area remains insufficient [184, 187, 188].

#### Hydrogen sulfide (H2S)

H2S derived from the gut microbiota is a key regulatory factor in the formation of blood vessels and lymphatic vessels. H2S directly acts on endothelial cells to induce the activation of the nitric oxide (NO) synthase/NO signaling pathway, promoting the development of cerebral blood vessels in rats [189]. H2S promotes endothelial cell proliferation, migration, and tube formation, demonstrating an angiogenic effect in rat wound healing and hind limb ischemia models, and exerts a protective effect through post-infarction vascular regeneration [190–193]. H2S regulates the development of various diseases, including hypertension and atherosclerosis, by influencing vascular tension [194]. H2S upregulates VEGF expression through various angiogenic signaling pathways, such as the mitogen-activated protein kinase pathway, phosphoinositide-3-kinase pathway, NOS/NO pathway, signal transducer and activator of transcription 3 (STAT3) pathway, and adenosine triphosphate (ATP)-sensitive potassium (KATP) channels, playing a role in cerebral vascular development, diabetic wound healing, refractory secondary lymphedema and physiological angiogenesis during pregnancy [195].

H2S promotes the formation of tumor blood vessels and lymphatic vessels and affects tumor metastasis by activating various signaling pathways [196–201]. Under certain conditions, H2S may also exert an inhibitory effect on tumor angiogenesis. For example, the H2S donor GYY4137 has been shown to inhibit tumor angiogenesis by disrupting the STAT3 signaling pathway in hepatocellular carcinoma, and another H2S donor, diallyl sulfide (DAS), has been confirmed in mouse models [202, 203]. This may be related to the concentration of H2S; at low concentrations, H2S appears to stimulate tumor angiogenesis and progression by activating the EGFR/ERK/MMP-2 signaling cascade. In contrast, at higher concentrations, H2S regulates the PTEN/AKT pathway and exerts an inhibitory effect on tumor angiogenesis [204].

#### Nitric oxide (NO)

Intestinal flora reduce nitrate to nitrite and produce NO and ammonia under physiological conditions [205]. NO derived from gut microbiota influences the generation of blood vessels and lymphatic vessels through various mechanisms, including regulating vascular tone and permeability, promoting endothelial cell proliferation, migration, and lumen formation [206–208]. These effects are significant for maintaining normal physiological functions and for the repair of blood vessels and lymphatic vessels in disease states. For instance, in a mouse model of cirrhosis and portal hypertension, FMT alleviates gut microbiota dysbiosis, decreases eNOS phosphorylation, reduces mesenteric angiogenesis, and mitigates collateral circulation in cirrhosis [209]. Furthermore, the optimization of gut microbiota ecology and the upregulation of NO production within the intestines contribute to the alleviation of ischemia in the hind limbs of diabetic mice [210]. NO can promote VEGF-induced tumor angiogenesis [211], induce MMP expression [212, 213], and enhance the invasion, migration of tumor cells, and the tube formation ability of endothelial cells. Studies have shown that NO can also normalize tumor blood vessels, which is beneficial for the delivery of chemotherapy drugs [214].

It is worth noting that current research has well elucidated the roles of H2S and NO in developmental angiogenesis and pathological angiogenesis. However, there are few studies that incorporate the role of H2S and NO as metabolites of gut microbiota. In other words, there is still a lack of observation on the effects of H2S and NO on developmental and pathological angiogenesis from the perspective of gut microbiota and its metabolites. However, it is undeniable that the human gut microbiota is the largest source of H2S. In germ-free mice, the levels of free hydrogen sulfide (H2S) in plasma and gastrointestinal tissues are significantly lower compared to conventionally raised mice. Additionally, the activity of cystathionine γ-lyase (CSE) in multiple tissues of germ-free mice is significantly reduced, while the levels of cysteine in the tissues are significantly elevated [215].

### The interaction between diet and gut microbiota affects angiogenesis in diseases

#### Polyphenol compound

In the process of absorption and metabolism of polyphenolic compounds in the human body, most of them (about 90%) are not absorbed by the small intestine but instead enter the colon, where they are acted upon by the gut microbiota, producing bioactive metabolites that may have an impact on human health [216]. Research indicates that polyphenolic compounds may influence angiogenesis and lymphangiogenesis by regulating the function, proliferation, and migratory ability of endothelial cells, as well as their anti-inflammatory effects [217–219]. In addition, polyphenols regulate the gut microbiota, affecting the production of TMAO [220].

Polyphenols can regulate the expression of VEGF in tumor tissues, affecting angiogenesis. For example, green tea catechin (–)-epigallocatechin gallate (EGCG) [221] and black tea metabolite theaflavin-3,3ʹ-digallate (TF3) [223] downregulate VEGF expression and inhibit tumor angiogenesis by suppressing the PI3K/AKT/mTOR/HIF1α signaling pathway. The combination of curcumin and EGCG downregulates IL-8 expression and inhibits the inflammatory response in the tumor microenvironment of colon cancer by suppressing the JAK/STAT3 signaling pathway [223]. This may be related to the regulation of gut microbiota composition and the production of metabolites by polyphenols [224, 225], this still requires further research to confirm.

#### Polyunsaturated fatty acids (PUFAs)

The intake of polyunsaturated fatty acids in the diet can affect the composition of the gut microbiota [226], which in turn influences the production of other gut microbial metabolites, impacting the host.For example, ω−6 polyunsaturated fatty acids, such as arachidonic acid (AA), can increase the abundance of *Gram-negative bacteria* in the gut [227, 228], triggering the TLR4/MYD88 signaling pathway and catalyzing the conversion of FADS1-AA to prostaglandin E2 (PGE2), which promotes angiogenesis and tumor growth [229, 230]. However, in vitro experiments have not shown a significant tumor-promoting effect of AA, indicating that its role in tumor progression may be regulated by the gut microbiota. A diet rich in ω−3 polyunsaturated fatty acids may increase the abundance of bacteria that produce short-chain fatty acids (SCFAs) [231], reduce the abundance of bacteria that produce trimethylamine (TMA) [232] and lipopolysaccharides (LPS) [233], and increase the abundance of probiotics.

## Gut pathogenic bacteria are related to angiogenesis in disease

The interaction between the gut microbiota and pathogens is crucial for preventing pathogen colonization and infection. For example, the microbiota inhibits the growth of pathogens by producing antimicrobial peptides and competing for nutrients [234]. In disease states, changes in the biofilm phenotype may cause originally symbiotic bacteria to become pathogenic symbionts. This can promote the occurrence and development of diseases by invading the mucus barrier, adhering to intestinal epithelium, crossing intestinal cells, and activating various pro-inflammatory pathways. In IBD and colorectal cancer, the best-characterized bacterial commensals that escape from a disturbed microbiota biofilm and turn into pathobionts include Adhering Invasive *E. coli (AIEC)*, *Bacteroides fragilis*, *Enterococcus faecalis*, and *Fusobacterium nucleatum* [235]. Regarding the colonization of pathogens, the body's gut microbiota has a complex defense network, including colonization resistance, competitive inhibition, nutrient competition, and anaerobic environments. When these defense mechanisms are disrupted, such as through the use of antibiotics or dietary changes, pathogens have the opportunity to grow to high levels, leading to the onset of disease.

Clostridium perfringens influences angiogenesis through the toxins it produces [236]. For instance, Clostridium perfringens type C generates beta-toxin (CPB), which initially binds to endothelial cells in the intestine and forms oligomeric pores on the cell surface [237]. This process results in vascular necrosis and disrupts the intestinal barrier function [61].The C3 type of botulinum toxin hydrolyzes a portion of C3b, engaging with Rac1 (Ras-related C3 botulinum toxin substrate 1) and resulting in its inactivation and the inhibition of Rac1 GTPase activity [238–240]. This disruption of the Rac1 signaling pathway results in the obstruction of blood and lymphatic vessel formation, which is significant in the context of tumor growth and metastasis, body development, and wound healing [241–245]. The amino acid peptide p28, derived from azurin released by *Pseudomonas aeruginosa*, non-competitively inhibits the phosphorylation of vascular endothelial growth factor receptor 2 (VEGFR2) and its downstream targets FAK (focal adhesion kinase), AKT (protein kinase B), and bFGF (basic fibroblast growth factor). This usually inhibits the movement and migration of HUVECs before the relocalization of the cytoskeleton (F-actin), focal adhesions (FAK and paxillin), and the intercellular adhesion protein PECAM-1 [62, 246]. High-affinity anthrax toxin receptor (ATR) ligands, such as PA and PASSSR (a mutant form of PA), exhibit high affinity for binding to endothelial cell capillary morphogenesis gene 2 (CMG2) and tumor endothelial marker 8 (TEM8), show competitive inhibitory effects, thereby inhibiting the angiogenic effects of VEGF and bFGF [247]. This anti-angiogenic effect is mediated by CMG2 [248–250]. Furthermore, the combination of lethal factor (LF) and PA, which forms anthrax lethal toxin (LeTx), has the capacity to impede the activation of the Erk1/2, p38, and JNK pathways by obstructing the MAPK pathway [251–253]. This inhibition results in the downregulation of pro-angiogenic cytokines, including VEGF and IL-8, in tumor and endothelial cells. This, in turn, suppresses angiogenesis and lymphangiogenesis [63]. Gut pathogenic bacteria can influence angiogenesis and lymphangiogenesis through diverse mechanisms, signaling pathways, and metabolites. These effects can facilitate bacterial survival and dissemination within the host. Conversely, the metabolites produced by these bacteria hold significant potential for development into therapeutic agents targeting angiogenesis and lymphangiogenesis.

Another pertinent example is *Helicobacter pylori*, a bacterium that predominantly colonizes the stomach. Research has demonstrated that *H. pylori* infection not only impacts the gastric microbiota but also induces alterations at both the phylum and genus levels within the intestinal microbiota [254]. Furthermore, *H. pylori* infection may exert an indirect influence on angiogenesis by enhancing the secretion of gastric acid and gastrin, altering the permeability of the intestinal mucosa, and modulating the gut microecological environment through the action of virulence factors such as CagA (cytotoxin-associated protein) and VacA (vacuolating cytotoxin-associated protein) [255–257].

*H. pylori* has been demonstrated to significantly elevate the expression of the inflammatory cytokine IL-8, stimulate the proliferation of lymphatic endothelial cells, and enhance the expression of LYVE-1, thereby facilitating the formation of both blood vessels and lymphatic vessels [64, 258–260]. Furthermore, *H. pylori* infection activates the p38/ATF-2 signaling pathway, which promotes the expression of cyclooxygenase-2 (COX-2). The resultant production of prostaglandin E2 (PGE2) can subsequently upregulate the expression of VEGF and B-cell lymphoma 2 (Bcl-2), thereby aiding in the development of vascular and lymphatic structures [261–266]. *H. pylori* infection has the potential to impede angiogenesis, resulting in a protracted healing process of peptic ulcers [267]. However, another study indicates that *H. pylori* infection activates the mitogen-activated protein kinase-extracellular signal-regulated kinase 1/2 (MAPK-ERK 1/2) pathway in gastric epithelial cells, resulting in the sustained expression of hypoxia-inducible factor 1-alpha (HIF-1α) and elevated levels of vascular endothelial growth factor (VEGF) under normoxic conditions, which in turn stimulates angiogenesis. Proton pump inhibitors (PPIs) effectively inhibit the phosphorylation of MAPK-ERK 1/2, thereby mitigating *H. pylori*-induced angiogenesis [65].

Furthermore, a substantial correlation exists between *H. pylori* infection and the advancement of gastric cancer [268]. Another study indicates that *H. pylori* infection triggers an inflammatory response in the gastric mucosa, promoting the expression of COX-2. This stimulates gastric epithelial cells to secrete the pro-angiogenic factor VEGF via the COX-2/Wnt/β-catenin pathway (Fig. 2), thereby facilitating the progression of gastric cancer [66]. In the context of mucosa-associated lymphoid tissue (MALT) lymphoma, *H. pylori* infection facilitates the formation of both blood vessels and lymphatic vessels within MALT lymphoma tissues via the VEGF and Flt pathways [269–273]. Inhibiting these receptors could be a promising strategy for the treatment of MALT lymphoma [42, 44–46, 274, 275].

## Prospect

**Fig. 3:**
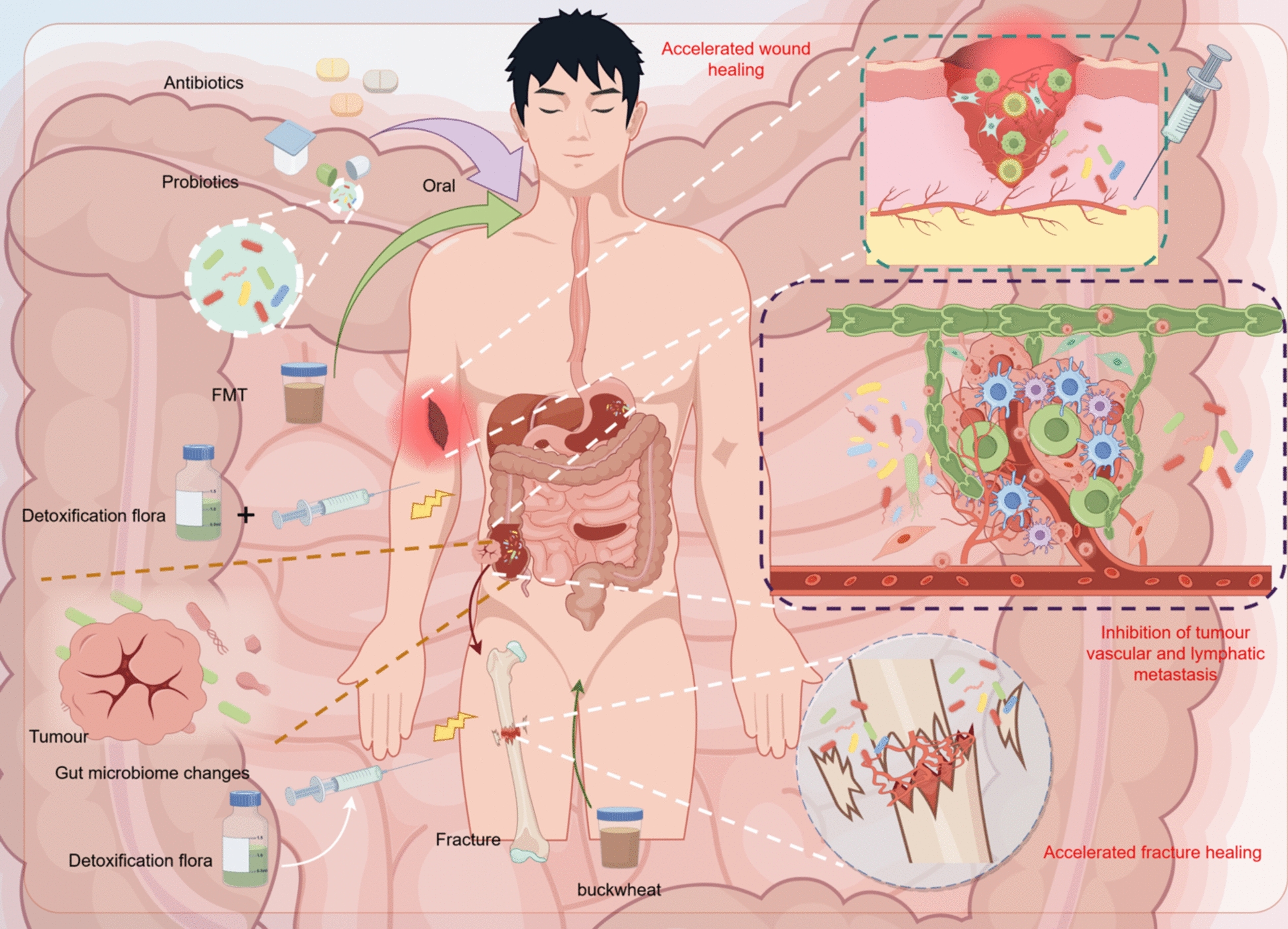
Influencing the composition of the intestinal flora by means of oral antibiotics, probiotics and FMT affects tumour angiogenesis and lymphangiogenesis. In addition, inactivation of flora by local injection may be a potential therapeutic strategy to promote fracture and wound healing

As shown in Fig. 3, the current main methods for regulating gut microbiota include fecal microbiota transplantation (FMT) and probiotics. FMT has shown good efficacy in treating inflammatory bowel disease (IBD) and irritable bowel syndrome (IBS) [276, 277]. A randomized controlled trial confirmed that encapsulated FMT is effective for recurrent Clostridium difficile infection (CDI) [278, 279]. FMT indirectly influences angiogenesis and lymphangiogenesis by regulating the metabolic byproducts of gut microbiota, such as SCFAs and TMAO. Probiotics can participate in immune regulation by inducing the differentiation of immune cells, interfering with the release of inflammatory factors, repairing the intestinal barrier, and regulating the gut microbiome and its metabolites [280–283]. They play a role in the treatment of IBD and IBS, as well as enhancing the efficacy of tumor immunotherapy [283–285]. Additionally, probiotic metabolites such as SCFAs and indole derivatives can also influence the occurrence and development of diseases.These methods have shown great potential in treating diseases, although the best strategy for "precisely" regulating gut microbiota is still not completely clear [286]. Improper use of antibiotics may disrupt the balance of gut microbiota, affect the levels of gut microbial metabolites such as TMAO, and impact host health [287, 288]. Analogous to fecal microbiota transplantation (FMT), engineered probiotics that employ gene therapy for the treatment of tumors are exhibiting promising therapeutic efficacy [289]. For instance, Wei et al. genetically modified Bifidobacterium longum to produce Tumor Vasculature Inhibitor (Tumstatin, Tum), which effectively suppressed the proliferation of tumor endothelial cells and triggered apoptosis in tumor cells within the CT26 tumor-bearing murine model [290]. In summary, improving gut microbiota and metabolic products through methods such as FMT and oral probiotics is still in the exploratory stage. There is currently no conclusion regarding the suitable populations and application dosages, and further research is needed. Diet not only regulates the composition of gut microbiota, but the metabolic products of gut microbiota can directly or indirectly affect angiogenesis and the development of cardiovascular diseases. For example, a high-fat diet can lead to increased expression of TMAO, while increasing dietary fiber intake may influence the production of SCFAs. Therefore, dietary interventions to regulate gut microbiota may provide new strategies for the prevention and management of cardiovascular diseases.

## Conclusion

This review has summarized how the gut microbiota and its metabolic products influence the formation of blood and lymphatic vessels through various signaling pathways, including the regulation of VEGF expression, modulation of inflammatory effects, interference with tumor immune responses and EMT (Epithelial-Mesenchymal Transition), the AKT signaling pathway, the Toll-like receptors (TLRs) signaling pathway, the Aryl Hydrocarbon Receptor (AHR) and Pregnane X Receptor (PXR) signaling pathways, and the Lysophosphatidic Acid (LPA) signaling pathway. Clarifying the impact of the gut microbiota and its metabolic products on angiogenesis and lymphangiogenesis, and by intervening in the abundance of the gut microbiota and the production of its metabolic products, it is possible to affect the generation of blood and lymphatic vessels, with the aim of preventing and treating diseases, especially in the field of cancer therapy, where we speculate there will be promising applications. However, current interventions for the gut microbiota are limited, and further research is needed to explore the feasibility of this therapeutic strategy. Additionally, this review also highlights the important role of diet in disease prevention and treatment; certain foods metabolized by the gut microbiota may affect the formation of blood and lymphatic vessels, thereby influencing the occurrence and progression of diseases. Different individuals have different susceptibilities to diseases, and the compositional differences in gut microbiota among individuals may be one of the factors affecting their different susceptibilities to various diseases and different responses to treatment plans. Understanding the role of different gut microbiota and their metabolic products in the body's angiogenesis and lymphangiogenesis will help to achieve more personalized medical interventions.

## Data Availability

No datasets were generated or analysed during the current study.

## Abbreviations

*HP*: *Helicobacter pylori*
BAI1: Brain-specific angiogenesis inhibitor 1
MALT: Mucosa-associated lymphoid tissue type
MAPK: Mitogen-activated protein kinase
ERK: Extracellular regulated protein kinases
HIF-1: Hypoxia inducible factor-1
VEGF: Vascular endothelial growth factor
PPIs: Proton pump inhibitors
IL-8: Interleukin-8
LYVE-1: Lymphatic vessel endothelial receptor-1
CXCR2: C-X-C motif chemokine receptor 2
COX-2: Cyclooxygenase 2
PGE2: Prostaglandin E2
p38/ATF-2: P38/activating transcription factor-2
BCL-2: B-cell lymphoma 2
Akt: Protein kinase B
CRC: Colorectal cancer
UC: Ulcerative colitis
Twist1: Twist family bHLH transcription factor 1
EMT: Epithelial–mesenchymal transition
GVB: Gut vascular barrier
PMN: Pre-metastatic niches
TLR4: Toll-like receptor 4
LPS: Lipopolysaccharide
NF-κB: Nuclear factor-kappa B
FOXC2: Forkhead box C2
DLL4: Delta-like 4
BMP-9: Bone morphogenetic protein-9
ALK: Anaplastic lymphoma kinase
*F. nucleatum*: *Fusobacterium nucleatum*
MYD88: Myeloid differentiation primary response 88
RAS: Rat sarcoma
TNF-α: Tumor necrosis factor-alpha
IL-6: Interleukin-6
IL-1β: Interleukin-1 beta
BFT: *Bacteroides fragilis* Toxin
TCF: T-cell factor
c-myc: Avian myelocytomatosis viral oncogene homolog
FGF: Fibroblast growth factor
HK2: Hexokinase 2
PSA: Polysaccharide A
TLR2: Toll-like receptor 2
CCR5: C–C chemokine receptor 5
BV: Bilirubin
mTOR: Mechanistic target of rapamycin
*KH2*: *Heat-killed Enterococcus faecalis*
bFGF: Basic fibroblast growth factor
TGF: Transforming growth factor
*LGG*: *Lactobacillus rhamnosus GG*
FPR1: Formyl peptide receptor 1
*B.P*: *Bacillus polyfermenticus*
HIMEC: Human intestinal microvascular endothelial cell
PDGF-BB: Platelet-derived growth factor-BB
CAC: Colitis-associated colorectal cancer
CTL: Cytotoxic T lymphocytes
MLN: Mesenteric lymph nodes
FAK: Focal adhesion kinase
ATR: Anthrax toxin receptor
CMG2: Capillary morphogenesis gene 2
TEM8: Tumor endothelial marker 8
LF: Lethal factor
LeTx: Lethal toxin
JNK: C-Jun N-terminal kinase
LCA: Lithocholic acid
GSK: Glycogen synthase kinase
UDCA: Ursodeoxycholic acid
SOCS: Suppressor of cytokine signaling
STAT: Signal transducers and activators of transcription
VCAM: Vascular cell adhesion molecule
TMAO: Trimethylamine N-oxide
CKD: Chronic kidney disease
SCFAs: Short-chain fatty acids
LEC: Lymphatic endothelial cell
CPT1A: Carnitine palmitoyltransferase 1A
SMAD: SMA and MAD related domain proteins
ALCAR: Acetyl-l-carnitine
LPA: Lysophosphatidic acid
ICAM: Intercellular adhesion molecule
HFD: High-fat diet
YAP/TAZ: Yes-associated protein/transcriptional coactivator with PDZ-binding motif
PPAR: Peroxisome proliferator-activated receptor
VCAM: Vascular cell adhesion molecule
NRP: Neuropilin
I3AA: Indole-3-acetic acid
IAld: Indole-3-aldehyde
AHR: Aryl hydrocarbon receptor
TF3: Theaflavin-3,3ʹ-digallate
PUFAs: Polyunsaturated fatty acids
LPS: Lipopolysaccharide
TMA: Trimethylamine
AA: Arachidonic acid
AIEC: Adherent-invasive Escherichia coli
PGE2: Prostaglandin E2
H2S: Hydrogen sulfide
Ang-1: Angiopoietin-1
DAS: Diallyl sulfide
NO: Nitric oxide
